# Academic Detailing as a Health Information Technology Implementation Method: Supporting the Design and Implementation of an Emergency Department–Based Clinical Decision Support Tool to Prevent Future Falls

**DOI:** 10.2196/52592

**Published:** 2024-04-18

**Authors:** Hanna J Barton, Apoorva Maru, Margaret A Leaf, Daniel J Hekman, Douglas A Wiegmann, Manish N Shah, Brian W Patterson

**Affiliations:** 1 BerbeeWalsh Department of Emergency Medicine University of Wisconsin-Madison Madison, WI United States; 2 Department of Information Services UW Health Madison, WI United States; 3 Department of Industrial and Systems Engineering University of Wisconsin-Madison Madison, WI United States

**Keywords:** emergency medicine, clinical decision support, health IT, human factors, work systems, SEIPS, Systems Engineering Initiative for Patient Safety, educational outreach, academic detailing, implementation method, department-based, CDS, clinical care, evidence-based, CDS tool, gerontology, geriatric, geriatrics, older adult, older adults, elder, elderly, older person, older people, preventative intervention, team-based analysis, machine learning, high-risk patient, high-risk patients, pharmaceutical, pharmaceutical sales, United States, fall-risk prediction, EHR, electronic health record, interview, ED environment, emergency department

## Abstract

**Background:**

Clinical decision support (CDS) tools that incorporate machine learning–derived content have the potential to transform clinical care by augmenting clinicians’ expertise. To realize this potential, such tools must be designed to fit the dynamic work systems of the clinicians who use them. We propose the use of academic detailing—personal visits to clinicians by an expert in a specific health IT tool—as a method for both ensuring the correct understanding of that tool and its evidence base and identifying factors influencing the tool’s implementation.

**Objective:**

This study aimed to assess academic detailing as a method for simultaneously ensuring the correct understanding of an emergency department–based CDS tool to prevent future falls and identifying factors impacting clinicians’ use of the tool through an analysis of the resultant qualitative data.

**Methods:**

Previously, our team designed a CDS tool to identify patients aged 65 years and older who are at the highest risk of future falls and prompt an interruptive alert to clinicians, suggesting the patient be referred to a mobility and falls clinic for an evidence-based preventative intervention. We conducted 10-minute academic detailing interviews (n=16) with resident emergency medicine physicians and advanced practice providers who had encountered our CDS tool in practice. We conducted an inductive, team-based content analysis to identify factors that influenced clinicians’ use of the CDS tool.

**Results:**

The following categories of factors that impacted clinicians’ use of the CDS were identified: (1) aspects of the CDS tool’s design (2) clinicians’ understanding (or misunderstanding) of the CDS or referral process, (3) the busy nature of the emergency department environment, (4) clinicians’ perceptions of the patient and their associated fall risk, and (5) the opacity of the referral process. Additionally, clinician education was done to address any misconceptions about the CDS tool or referral process, for example, demonstrating how simple it is to place a referral via the CDS and clarifying which clinic the referral goes to.

**Conclusions:**

Our study demonstrates the use of academic detailing for supporting the implementation of health information technologies, allowing us to identify factors that impacted clinicians’ use of the CDS while concurrently educating clinicians to ensure the correct understanding of the CDS tool and intervention. Thus, academic detailing can inform both real-time adjustments of a tool’s implementation, for example, refinement of the language used to introduce the tool, and larger scale redesign of the CDS tool to better fit the dynamic work environment of clinicians.

## Introduction

### Background

New technologies incorporating machine learning–derived content into clinical decision support (CDS) have the potential to bring transformative improvements to clinical care [1-3]. Identifying high-risk patients who merit referral for preventative care services has historically required cumbersome screening, but now can be rapidly completed by risk prediction algorithms that consider the patient’s entire electronic health record (EHR) [4-6]. By incorporating machine learning–derived content, clinicians’ decision-making can be augmented by insights that may otherwise go unnoticed. Yet the potential benefits of these CDS tools will only be realized when they are designed to fit the clinical contexts in which clinicians work [3,7]. Health information technologies (HITs), including CDS tools, that fail to fit clinicians’ decision-making processes and workflows are unlikely to be adopted and even risk increasing clinician burden and burnout [8-10].

However, even technologies that are designed using today’s best usability guidance [11] often fail to fit the clinical context upon initial implementation [12,13]. As health systems continue to evolve in response to emergent patient needs and expectations (eg, COVID-19 and its aftermath), regulatory requirements, and staffing challenges, CDS tools are being implemented in increasingly sensitive and complex environments. While implementation science frameworks consider a variety of contextual factors [14-16] and some methods exist for assessing and identifying them [17,18], there is a gap in methods for rapidly identifying contextual factors immediately postimplementation—when it may be easiest to respond to and redesign for emergent barriers to the technology’s use, safety, and effectiveness [19].

One method that has the potential to be adapted to rapidly identify contextual factors influencing the implementation of HIT is *academic detailing*. A repurposing of pharmaceutical sales representatives’ tactics, academic detailing is defined as a “personal visit by a trained person to health professionals in their own settings” [20]. The goal of these personal visits is to improve care quality and patient outcomes by promoting evidence-based practice through focused clinician education [21]. As an implementation method, academic detailing can be conceptualized as a combination of 3 Expert Recommendations for Implementing Change (ERIC; also known as Evidence-based Recommendations for Implementing Change) strategies: auditing and providing feedback, conducting educational outreach visits, and practice facilitation [14]. The method’s attention to the specific contexts in which clinicians make decisions—both by conducting visits in situ and by discussing barriers to and strategies for making evidence-based decisions—may present a unique opportunity to not only promote the use of a newly implemented HIT but also identify contextual factors influencing its initial implementation.

### Study Objective

We propose the use of academic detailing as a method for achieving two goals in the implementation of an emergency department (ED)–based CDS tool to prevent future falls: (1) ensuring the correct understanding of the tool and its evidence base and (2) identifying contextual factors influencing the tool’s initial implementation. As part of a long-term goal of assessing academic detailing for achieving these 2 aims, the objective of this study was to assess academic detailing through an analysis of the resultant qualitative data.

## Methods

### Study Context and Setting

This study was conducted at a large academic medical center located in the Midwestern United States. The associated ED, a level 1 trauma center, treats over 60,000 patients per year. The CDS tool being evaluated is intended to facilitate both screening for outpatient fall risk among older adults presenting to the ED and the referral to a fall prevention clinic for those patients at high risk. Our research team developed an outpatient fall risk prediction algorithm from EHR data and, in concert with our partner health system, designed and implemented a CDS tool to use the algorithm that went live in July 2020 [22,23]. In November 2020, the CDS was updated such that it enforced a “hard stop” in the clinician’s workflow and required them to interact with it.

Upon arrival to the ED, all patients aged 65 years and older with an in-system primary care provider are assessed for fall risk algorithmically based on their extant EHR data. For eligible patients who are at high risk for falls, during the discharge process, an interruptive CDS alert is shown to clinicians, which informs them of the patient’s risk factors and expedites the placement of a referral order to a mobility and falls clinic, an evidence-based preventive intervention. Patients who are referred are informed both by the nursing staff and in writing and are contacted to schedule an appointment by scheduling staff in the days following their ED visit. This intervention has been described in more detail elsewhere [23,24].

### Study Design

**Figure 1:**
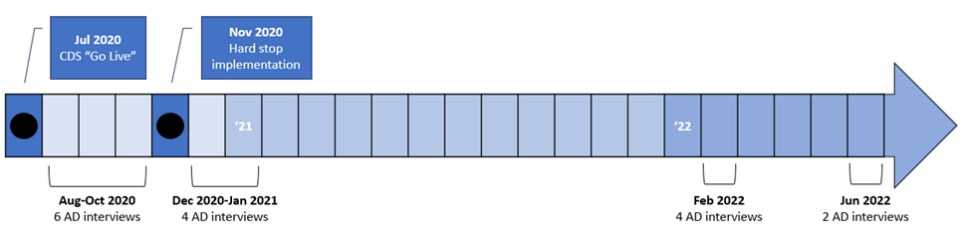
Timeline of clinical decision support implementation and academic detailing interviews. AD: academic detailing; CDS: clinical decision support.

To assess academic detailing as a method for simultaneously achieving the goals of ensuring the CDS was understood and identifying contextual factors influencing its implementation, we used a qualitative approach. We conducted 16 semistructured academic detailing interviews with emergency medicine resident physicians (n=10) and advanced practice providers (n=6) who had previously encountered our CDS tool in practice, that is, within the last month. All interviews took place between August 2020 and June 2022, with 6 of the 16 interviews occurring prior to the implementation of the CDS hard stop (Figure 1). We purposively selected a range of participants based on how frequently they responded to the CDS. The academic detailing interviews were led by an intervention expert (AM) who had a comprehensive understanding of the CDS tool and thus was able to identify and correct any misconceptions about the tool and its use—a critical aspect of effective academic detailing [21].

### Study Procedure

Interviews were roughly 10 minutes long and took place over the phone or in person while the clinician was on shift. The intervention expert used an interview guide developed using the critical incident technique, which asks the participants to mentally put themselves in the moment they first saw the tool in the EHR [25]. The interview guide (Multimedia Appendix 1) contained questions such as “How and when did you see the tool initially? What was your reaction? How did you make the decision to refer the patient or not?” Additionally, comments made by a participant that suggested an incomplete or inaccurate understanding of the tool were addressed by the intervention expert (eg, “the tool refers patients to the Mobility and Falls clinic, not the Faint and Falls clinic”). Phone interviews were transcribed in real time, while notes were taken during in-person interviews and then written out immediately after.

### Ethical Considerations

This study was reviewed and determined to be exempt by the UW-Madison Health Sciences IRB (ID# 2020-1100). Participants were not compensated, and data were deidentified for analysis.

### Data Analysis

We conducted an inductive, team-based content analysis [26,27]. Two researchers (AM and MAL) began by independently reviewing and coding 4 interviews, line-by-line, to identify factors that influenced clinicians’ decision-making. The researchers then met to compare and refine codes until there was agreement. This process continued iteratively until all interviews were coded; the resultant codebook contained 31 codes and subcodes (eg, patient risk factors and clinician communication). Another researcher (HJB) generated categories of factors that influenced clinicians’ referral or nonreferral from the codes through a process of organizing similar and dissimilar codes, periodically incorporating feedback from the research team, until there was agreement.

## Results

### Overview

We identified five categories of factors that impacted clinicians’ use of the CDS: (1) aspects of the CDS tool’s design, for example, its features, usability, and how it fits in the clinician’s workflow; (2) clinicians’ understanding (or misunderstanding) of the CDS or referral process; (3) the busy nature of the ED environment; (4) clinicians’ perceptions of the patient and their associated fall risk; and (5) the opacity of the referral process. Table 1 organizes the identified factors by these categories, including a description of the type of clinician education that was done during the academic detailing interviews.

**Table 1:** Factors impacting clinicians’ CDS^a^ use identified through academic detailing and clinician education done during academic detailing interviews. Positive and negative factors are indicated by the +, +/–, and – symbols.

Categories of factors impacting clinicians’ CDS use through academic detailing	Factors impacting clinicians’ CDS use identified through academic detailing	Clinician education done during academic detailing interviews
Aspects of the CDS tool’s design	[+] CDS is simple. [+] CDS requires minimal input from the clinician. [+] CDS automatically identifies a high-risk patient and prompts care that the clinician would not otherwise have considered. [+/–] CDS enforces a hard stop in the clinician’s workflow. [+/–] CDS alert fires while the clinician is completing discharge in the EHR^b^.	Discussed why the CDS alert fires when it does and the potential benefits and challenges of it firing at a different point in the clinician’s workflow.
Clinicians’ understanding of the CDS or referral process	[–] Clinician confuses the geriatric mobility and falls clinic with the faint and falls clinic. [–] Clinician believes only patients being seen for a fall are appropriate referrals. [–] Clinician believes referring the patient will be cumbersome, ie, require written justification. [+] Clinician is familiar with the concept of the CDS from an organizational stakeholder’s communication.	Clarified which clinic the referral goes to. Clarified that referral is appropriate preventative care for patients regardless of their presenting problem. Demonstrated how simple and quick it is to place a referral via the CDS.
Busy nature of the ED^c^ environment	[+/–] A busy ED environment.	
Clinicians’ perceptions of the patient and their associated fall risk	[+/–] Clinicians’ agreement with the CDS’s assessment of the patient’s fall risk. [–] Clinicians’ perception of the patient’s openness to, need for, or benefit from the intervention.	Demonstrated where in the CDS to find the reasons the patient is being flagged as high risk. Stressed the potential benefits of a successful referral for both the patient and health system.
Opacity of the referral process	[–] Clinicians lack clarity on where the referral goes once it is sent. [–] Clinicians are uncertain about who should communicate with the patient about the referral, ie, themselves or a nurse. [+/–] Clinicians (do not) have the information necessary for counseling patients on what to expect from the referral and why they are being referred.	Clarified which clinic the referral goes to. Clarified the importance of counseling patients on the referral and demonstrated where in the CDS to access information to support counseling patients on the referral. Demonstrated where in the CDS to find the reasons the patient was flagged as high risk.

^a^CDS: clinical decision support.

^b^EHR: electronic health record.

^c^ED: emergency department.

### Aspects of the CDS Tool’s Design

The first category of factors that influenced clinicians’ use of the CDS was those that related to the design of the CDS tool. Many clinicians described the CDS as user friendly or easy to use, citing the limited number of clicks required and that the CDS did not require the clinician to enter any text. Further, clinicians found that the automatic nature of the CDS tool supported them in providing appropriate care that they otherwise would not have considered:

I appreciated how it fired on its own. I wasn’t even thinking about falls in the patient because he came in for [condition], not a fall. When it fired, I realized he was a great candidate, but it wasn’t something I thought about prior.

Participant 12

Further, clinicians described how the CDS integration into their workflow impacted their use of the CDS. For one, the CDS enforced a “hard stop,” requiring the clinician to interact with it. While clinicians’ feelings on the hard stop varied from being annoyed to finding it valuable, it was only described as impacting CDS use during high-volume times in the ED, which is discussed further in the next section. Clinicians generally appreciated where the CDS fit into their workflow—upon discharging the patient in the EHR—such that it “doesn’t seem like an additional step” (participant 15). Some clinicians noted the timing of the CDS as being too late in the care process to have a personal discussion with their patient about the referral, for example, they are discharging their patient in the EHR after they have already done their final visit to the patient’s room. Thus, when clinicians saw the CDS alert after their final visit with the patient, they described being less likely to refer the patient because of the additional time necessary to go back to the room to discuss the referral. However, most clinicians thought the CDS alert was well situated in their workflow:

It just pops up at discharge. It’s just a click, the referral order is already filled out. It’s very easy to use. It adds maybe 20 seconds to the discharge process.

Participant 14

### Clinicians’ Understanding of the CDS and Referral Process

The second category of factors that influenced clinicians’ use of the CDS was clinicians’ understanding, or misunderstanding, of the CDS and the referral process. One such misunderstanding of the CDS was clinicians confusing a separate faint and falls cardiology Clinic with the actual target of the referral, the mobility and falls clinic, which specifically addresses geriatric falls. Consequently, 2 clinicians cited their nonreferral as due to the inappropriateness of the faint and falls clinic for their geriatric patient:

I think more of the syncope patients and possible cardiac or peripheral vertigo patients who don’t need to be admitted or are younger and are less high risk and are anxious about having syncopal episodes for the first time…. We want to get [them to] an outpatient visit so they’ll be more likely to follow up with the clinic and have [care] done. I don’t think of it as much who just have a mechanical fall.

Participant 1

Further, 6 clinicians described that they would not refer a patient who was being seen for another chief complaint, believing that only patients being seen for a fall are appropriate referrals.

Additionally, 3 clinicians expressed concerns about how cumbersome they believed the referral would be; however, their perception of the tool changed immediately once it was demonstrated that it only required 2 clicks. For example, participant 2 said:

The BPA would be less annoying if I knew I didn’t need to justify it. If I had known that’s all I had to do, I would have clicked [to accept the referral].

One clinician stated that they expected that the CDS would be cumbersome, that is, that they would have to write out the referral because they thought “[the CDS] would be like the other referrals” that they had come across in the EHR (participant 8).

On the other hand, 1 factor in this category that positively impacted clinicians’ use of the CDS was that the CDS was familiar to some clinicians given previous communication from an organizational stakeholder (ie, clinical champion). For example, participant 12 said:

When the CDS fired, I knew [clinical champion] sent an email about this. From my interactions with this patient, I thought [they were] at high risk of fall and knew [they would] benefit from it. That’s why I tried to place that consult.

### Busy Nature of the ED Environment

The third category influencing CDS use was the busy nature of the ED environment. Five clinicians described the ED environment as a factor impacting their CDS use, whereas at least 1 clinician explicitly said, “the ED environment wouldn’t affect whether or not I refer a patient” (participant 4). Clinicians varied in their description of the impact of the busy ED environment, ranging from “I would ignore the [CDS]” (participant 12) to “I just do the referral” (participant 14). Those clinicians who said that the ED environment increased their likelihood of referring the patient cited the CDS’s hard stop and a significant amount of text as associated factors that shaped their decision-making. While other clinicians described the amount of text in the CDS as a stressor, it was not otherwise described as influencing clinicians’ use of the CDS.

### Clinicians’ Perceptions of the Patient and Their Associated Fall Risk

The fourth category of factors influencing the use of the CDS was clinicians’ perceptions of the patient and their associated fall risk. Overall, most clinicians (10/16) agreed with the CDS’s assessment of the patient’s fall risk. One clinician said:

In general, my reaction has been “oh that kinda makes sense.” It was always kind of a surprise in the sense that I hadn’t really considered the risk of falls before, but it never seems outlandish that that was a potential concern.

Participant 3

A few clinicians described instances of being annoyed by the firing of the CDS when it seemed irrelevant and thus did not use it. Conversely, another clinician was frustrated when the CDS did not fire when they expected to see it. Clinicians described occasionally not referring patients because they appeared to be “independent and functional” (participant 11) or “generally active and stable” (participant 10), or because their fall was “strictly mechanical” (participant 7).

Further, clinicians’ perception of the patient’s openness to or need for intervention impacted their CDS use. One clinician described factoring their assessment of the patient’s openness to going to the mobility and falls clinic into their decision to refer the patient or not:

You can kind of get a vibe if someone is going to the doctor. If you tell them there’s another doctor you can see; if they don’t even want to talk to me, I doubt they’re going to go to another doctor. If it’s going to be useless, I don’t want to waste everyone’s time. I like to tell people and if they say I’m not going to that then I won’t refer. I think I dictate if I’m going to [refer the patient] based on the conversation.

Participant 8

Clinicians also described being more likely to refer patients who they perceived as having a greater need for the intervention. For example, a clinician said, “If the patient seems more anxious or [they] don’t have as good of a support system or advocate, I would refer them” (participant 1). Alternatively, clinicians also described assessing how the referral would fit into the patient’s care plan, for example, if the patient had an upcoming surgery, the clinician would opt not to refer the patient so as to not “throw an extra thing on top of them” (participant 6).

### Opacity of the Referral Process

The final category of factors influencing clinicians’ use of the CDS was the opacity of the referral process. One clinician described how they lacked clarity on where the referral goes once it is sent by saying, “it feels like I’m just sending the referral off to the void and I don’t know who they’re getting referred to” (participant 3). In contrast to a clinician confusing the mobility and falls clinic with the faint and falls clinic, discussed previously, this clinician was specifically pointing to the lack of feedback they received about how the process of referring a patient to the mobility and falls clinic unfolds over time. Consequently, another factor clinicians said impacted their use of the CDS was the ambiguity around who should communicate with the patient about the referral: themselves or a nurse. Clinicians described this factor as being more prominent if they had already spoken to the patient and thus referring the patient would require them to initiate another conversation with the patient themselves or “hope the nurse will tell the patient” (participant 1).

Finally, a few clinicians said they lacked the necessary information to counsel the patient—either about what to expect from the referral and the mobility and falls clinic or about the reasons the patient had been flagged as high risk for falls. One clinician suggested that having guidance on how to counsel the patient might make referring patients an easier choice:

I haven’t been really having detailed conversation about what this entails and what they should expect. In the moment I hadn’t quite seen a link on how to counsel patients on this referral…I do want to refer patients…I just wish I knew what to tell patients.

Participant 3

Clinicians also described lacking sufficient information to explain to patients why they were flagged by the CDS as high risk for falls. In particular, clinicians said this information would likely influence their referring of patients being seen for chief complaints other than a fall by making it easier to explain the referral to the patient.

### Clinician Education Done During Academic Detailing

During the academic detailing interviews, various misconceptions were addressed directly by the intervention expert through clinician education. First, 1 misconception described previously was the mistaken belief that a referral to the mobility and falls clinic would be inappropriate for people being seen for a chief complaint other than a fall. The intervention expert addressed this by clarifying that the CDS alert fires for any older adult being seen in the ED who is at high risk of falling in the future regardless of their presenting complaint, so barring any contraindications—for example, patient in hospice—it would be appropriate to refer the patient. The intervention expert also clarified, for clinicians who misunderstood, the correct target clinic of the referral (ie, the mobility and falls clinic). Generally, the intervention expert stressed the potential benefits of a successful referral for both the patient (eg, improved quality of life) and the health system (eg, reduced use).

Another misconception that was addressed via academic detailing was the perception that referring a patient would be too cumbersome. By demonstrating that accepting the CDS alert and placing a referral takes only 2 clicks, this misconception was promptly addressed. The intervention expert also demonstrated where in the CDS to access information to support counseling patients on the referral and where in the CDS to find the reasons the patient was flagged as high risk. Finally, for any clinicians who had issues with where the CDS alert fired in their workflow, the intervention expert discussed the reasons for the alert firing when it does and the potential benefits and challenges of it firing at a different time. Oftentimes, after discussion, the clinician had a new appreciation for the complexity of designing the CDS alert.

## Discussion

### Principal Findings

This study demonstrates the use of academic detailing for supporting the early implementation of HIT, allowing us to identify and begin to address factors that impacted clinicians’ use of the CDS while concurrently educating clinicians to ensure the correct understanding of the CDS tool and intervention. By bundling multiple ERIC strategies, academic detailing appears to be a promising method for providing timely feedback to improve HIT implementation.

### Addressing Contextual Factors Within Detailing Sessions

A key component of the academic detailing method is its emphasis on clinician education [21] which, in the context of our study, involves correcting clinicians’ misconceptions. For example, 1 misconception that we identified and addressed through clinician education was the mistaken belief that a referral to the mobility and falls clinic was only appropriate for people being seen for a fall. Given the nature of this CDS tool, that is, its ability to predict future risk, the impact of this misconception is that the opportunities to intervene in the routine care of high-risk patients being treated for other chief complaints would be missed. As participant 12 articulated, quoted in the “aspects of the tools design” results section, a particular value of the CDS tool is that it runs automatically, that is, does not require clinician initiation; thus, it can prompt the clinician to consider fall risk—and care to address that risk—that they may not have been considering previously. Embedding clinician education into academic detailing thus addressed a high-impact misconception with immediacy. However, it remains to be seen whether and how addressing these misconceptions translates to clinicians’ use of the CDS tool. Our future work will explore the impact of these academic detailing sessions on implementation incomes, for example, clinicians’ rates of referral and their acceptance of the tool.

Another important misconception to address within the academic detailing interviews was the perception that referring a patient would be too cumbersome. By demonstrating the simplicity of accepting the CDS alert and placing a referral, this misconception was promptly addressed which likely prevented its propagation. However, as described in the Results section, clinicians’ perceptions of the CDS tool are situated within the context of the existing EHR and thus are beholden to a broader understanding of how similar tools work (ie, a mental model) [28]. As such, clinicians’ responses to CDS alerts can be understood to be habitual, triggered by environmental cues [29]; therefore, solely addressing this misconception at the clinician level is unlikely to sustain CDS use over time. Altering clinicians’ mental models of CDS tools and the EHR warrants systems-level redesign.

The content of the clinician education that is included in academic detailing is paramount to its success in increasing the use of an intervention [21]. Previous literature also notes the importance of the relationship between the clinician and the person doing the clinician education [21,30]. For this study, the intervention expert who conducted the academic detailing interviews had extensive experience working with the ED staff and had developed a rapport with them. To carry out academic detailing in another setting, there may be initial relationship- and trust building to do to achieve the detailed results our intervention expert was able to capture. Yet, given their role as a researcher (vs a fellow clinician), there were potentially missed opportunities for educating clinicians on topics that would have been better received from a colleague. For example, the deeply entrenched custom of referring to many older adults’ community-based falls as being “mechanical,” a catch-all term for falls that does not have an emergent, addressable cause, is known to negatively affect care [31]. This could have potentially been addressed by a colleague; however, in this study, we did not address this clinician perception as it fell outside of the expertise of our intervention expert, that is, outside of the purview of the CDS tool and the referral it recommends.

### Addressing Contextual Factors via Redesign

The factors impacting clinicians’ use of the CDS point directly to opportunities to intervene in and improve the CDS implementation process (Textbox 1). As discussed previously, clinician education can be done immediately, within the academic detailing interview; however, the clinician education that had to be provided within the interview can inform the redesign of a better rollout (eg, addressing what are likely to be misconceptions up front). Future rounds of academic detailing should thus result in the need for less or different clinician education from the intervention expert.

**Potential approaches for intervening in the health information technology implementation process to improve clinical decision support acceptance and use.**

**Real time (within academic detailing interview)**

- Demonstrating the current capabilities and function of the tool, for example, how easy it is to place a referral, where to access information about why the patient was flagged as high risk, and information to support counseling the patient on the referral.
- Discussing why the clinical decision support tool works the way it does and the potential benefits and challenges of redesigning it.
- Clarifying how the referral works, where it goes, and who is an appropriate candidate for the intervention.
- Addressing problematic or harmful misconceptions, for example, that there is no role the emergency department can play in providing preventive care after “mechanical falls.”
- Discussing how successfully using the clinical decision support and placing a referral improves patient outcomes and health system outcomes, for example, by reducing future visits to the emergency department.

**Short term (quick fixes)**

- Attending regularly scheduled meetings with clinicians to remind them about the clinical decision support and clarify misconceptions about placing the referral.
- Associating the organizational stakeholder’s name or image with the clinical decision support.
- Adding the mobility and falls clinic information to the clinical decision support, that is, the phone number and location.

**Long term (adaptation and redesign)**

- Developing feedback mechanisms for clinicians to hear about successfully referred patients.
- Clarifying roles around patient communication, that is, what is communicated by the clinician versus the nurse, and designing the clinical decision support to support those roles.
- Reviewing clinical decision support tools for potential interaction effects, for example, 2 clinical decision support tools fire on similar populations and are likely to be confused.
- Providing talking points on what the patient can expect after discharge with respect to scheduling and going to an appointment with the mobility and falls clinic.
- Providing talking points that explain why patients being seen for issues other than falls may be referred.
- Personalizing the timing of the clinical decision support alert for clinicians who tend to talk to patients before completing the discharge in the electronic health record, for example, moving the clinical decision support alert earlier in clinicians’ workflow.

In the longer term, a variety of approaches could be used to address the factors we identified as impacting the clinicians’ use of the CDS. For one, reviewing the CDS tools that are currently implemented in overlapping clinical contexts could identify potential interactions with the newly implemented CDS. To avoid interaction effects, the new CDS could be redesigned to differentiate it from others, for example, to alert a more specific patient population or to have clear visual cues and messaging. Alternatively, a review of the CDS ecosystem may prompt the removal of underused or ineffective CDS tools. Recent research, while limited, suggests that health systems that optimize CDS alerts, that is, reduce unnecessary or less useful alerts, see improved CDS use [32]. Further, those effects are not limited to the optimized CDS but spread to other CDS in the system [32].

Other redesigns that would address factors identified through academic detailing could address the workflow integration of the CDS. For example, for the clinicians who typically talk to a patient before completing the discharge in the EHR, moving the firing of the CDS alert earlier in the clinical workflow may be warranted. Beyond considering the timing of the CDS, to achieve workflow integration as defined by Salwei et al [33,34], the design of the CDS should consider the dimensions of flow, scope of patient journey, and level. An example of such a redesign could be—in the case where the fall risk CDS alert would happen earlier in the clinician’s workflow—allowing the clinician to “snooze” the alert until the point at which they have discussed the mobility and falls clinic referral with the patient. This design would increase the chance that the clinician would see the CDS prior to speaking with the patient for the last time, which could promote more meaningful patient counseling on fall risk; however, this design could also have unintended consequences, which should be explored prior to broad implementation.

In designing for CDS use, it is important to remember that increased use does not always equate to increased *appropriate* use (ie, referrals for patients that are a good fit for the mobility and falls clinic intervention). Thus, the findings from academic detailing should also be considered in light of, and be used to design to support, successful teaming between the CDS tool and the clinician. A potential design to promote teamwork between the CDS tool and the clinician could be to include on the CDS a list of exclusion criteria for the mobility and falls clinic that the CDS tool is unable or poorly able to assess (eg, late-stage dementia). The clinician, then, when considering referring the patient would be alerted to where their clinical judgment is especially necessary.

### Work Systems Approach to Redesign

Given the breadth of potential redesign options and the challenge of prioritizing efforts to improve not only CDS use, but *appropriate* CDS use, it is pertinent to consider models that can hold and make sense of system complexity. One model that has proven to be valuable across a variety of health care domains and in supporting the design of technologies—the Systems Engineering Initiative for Patient Safety (SEIPS) model—conceptualizes the work of clinicians as happening in a *work system*, which invariably influences care processes and outcomes [35-37]. The SEIPS model, which synthesizes literature on job stress, job design, and health care quality [37,38], provides a theoretical foundation for understanding why the system is achieving certain outcomes and how the system may be redesigned to achieve alternative outcomes.

In a parallel analysis—presented elsewhere [39]—we found that the data we collected using the academic detailing method successfully mapped to the SEIPS model’s work system components, for example, the *people* who do the work, the *tasks* they complete, the *tools and technologies* they use, and the *physical* and *organizational environment* they work in. A key aspect of the SEIPS model is the conceptualization of balance—that work system components that negatively influence processes and outcomes (barriers) may be balanced by positive components (facilitators) [37,40]. Thus, through redesign efforts, we can either seek to address the work system barrier or enhance the work system facilitator. Applying a work systems approach to system redesign to address the factors we identified through academic detailing has the potential to result in more sustainable HIT implementation.

Beyond redesigning the CDS itself, as discussed in the previous section, redesigning the work system to clarify the process of referring a patient to the mobility and falls clinic may be essential to promoting the appropriate use of the CDS. This would require creating clarity around who should communicate and about what with the patient (ie, the referring clinician and the nurse). Further, creating transparency around the positive outcomes of past referrals to the mobility and falls clinic (ie, success stories) may promote trust in the referring clinicians that this is an action worth taking.

### Limitations

The following limitations of our study should be considered. First, the academic detailing method, as applied here, relies on the clinicians to report what they perceive as influencing their use of the CDS. However, it is possible that clinicians’ perceptions differ from what they actually do—a common challenge in understanding people’s work is the difference between “work as imagined” versus “work as done” [41]. Second, this study focuses on academic detailing around a specific CDS tool that produces an interruptive alert to which a clinician must respond that they agree or decline to refer the patient. It is possible that there are other considerations for CDS tools and HIT that operate differently from this study (eg, tools that require more in-depth information processing or that must be initiated by the clinician). Further, given this academic detailing method was applied in a live ED setting over nearly 2 years—including multiple waves of COVID-19—a variety of external factors may have contributed to clinicians’ use (and perception of their use) of the CDS. Finally, it is yet unclear how many rounds of academic detailing would be required to capture and address the majority of factors impacting the implementation of the HIT. Future research should explore the use of the academic detailing method over a broader range of the implementation process so that the effort and resources required to conduct the interviews are used most effectively.

### Conclusions

With HIT developing at rapid speeds, it is essential we develop methods to support its integration into the complex environments in which they will be used. From our initial study, it appears that academic detailing is a promising method for both promoting the correct understanding of a CDS tool and identifying contextual factors influencing its implementation. Thus, academic detailing can inform real-time adjustments of a tool’s implementation (eg, refinement of the language used to introduce the tool), and larger scale redesign of the CDS tool to better fit the dynamic work environment of clinicians.

## Appendix

Multimedia Appendix 1 Interview guide for academic detailing interviews.

## Abbreviations

CDS: clinical decision support
ED: emergency department
EHR: electronic health record
ERIC: Expert Recommendations for Implementing Change, also known as Evidence-based Recommendations for Implementing Change
HFE: human factors engineering
HIT: health information technology
SEIPS: systems engineering initiative for patient safety
