# Supplementary material for: Academic Detailing as a Health Information Technology Implementation Method: Supporting the Design and Implementation of an Emergency Department–Based Clinical Decision Support Tool to Prevent Future Falls
Source: JMIR Hum Factors. 2024 Apr 18;11:e52592. doi: 10.2196/52592 (PMC11066751; doi:10.2196/52592)
Supplement: Multimedia Appendix 1 [file humanfactors_v11i1e52592_app1.docx]

Appendix 1

**Interview guide for academic detailing interviews**

*Important: No identifying information about the provider or their patients should be recorded in the interview notes.* Questions are to be framed in a non-judgmental way. Interviewer will remind clinicians that the answers will be de-identified. [Clinician] will not know who participated and answers will in no way be tied to individual providers. Participation is voluntary. They may choose not to answer any questions they wish, and they can stop the interview at any time.

1. Thinking back to [date of patient discharge]… from what you remember, can you describe how and when you saw the CDS letting you know that one of your patients’ was at high risk of future falls?
2. Can you remember what your reaction was to seeing that alert?
3. Can you walk me through how you made the decision about whether or not to refer that patient to the Mobility and Falls Clinic?
   - What prompted you to make those choices?
4. Do you think the automated system accurately identified the patient as being high risk? (Why or why not)
5. What features of the screening and referral process made it easy to use?
6. Were there any things about the CDS process that you found frustrating? (What were they? What made it so frustrating?)
   - What would you like to see changed that would make that less frustrating?
7. How did this CDS compare with other types of BPAs you have received while working in the ED?
   - Note: use the term “BPA” (best practice alert) since clinicians are more familiar with it.
8. In general, what types of factors might prompt you to order a referral?
   - Characteristics of the patient?
   - Factors related to the ED/work environment? (temporary or regularly-occurring)?
9. What factors might cause you to *not* order a referral?
   - Characteristics of the patient?
   - Factors related to the ED/work environment? (temporary or regularly-occurring)
10. What types of patients do you think might benefit the most from this automated identification and referral process? The least?
11. Are there any other changes that you would want to see made that would improve ED clinician’s experience with or use of the CDS? (If so, what?)
12. Any additional comments?
